# Targeting the BspC-vimentin interaction to develop anti-virulence therapies during Group B streptococcal meningitis

**DOI:** 10.1371/journal.ppat.1010397

**Published:** 2022-03-22

**Authors:** Haider S. Manzer, Ricardo I. Villarreal, Kelly S. Doran

**Affiliations:** University of Colorado Anschutz Medical Campus, Department of Immunology and Microbiology, Aurora, Colorado, United States of America; University of California, San Francisco, UNITED STATES

## Abstract

Bacterial infections are a major cause of morbidity and mortality worldwide and the rise of antibiotic resistance necessitates development of alternative treatments. Pathogen adhesins that bind to host cells initiate disease pathogenesis and represent potential therapeutic targets. We have shown previously that the BspC adhesin in Group B *Streptococcus* (GBS), the leading cause of bacterial neonatal meningitis, interacts with host vimentin to promote attachment to brain endothelium and disease development. Here we determined that the BspC variable (V-) domain contains the vimentin binding site and promotes GBS adherence to brain endothelium. Site directed mutagenesis identified a binding pocket necessary for GBS host cell interaction and development of meningitis. Using a virtual structure-based drug screen we identified compounds that targeted the V-domain binding pocket, which blocked GBS adherence and entry into the brain *in vivo*. These data indicate the utility of targeting the pathogen-host interface to develop anti-virulence therapeutics.

## Author summary

Group B *Streptococcus* (GBS) is the leading cause of bacterial neonatal meningitis. GBS utilizes a surface adhesin known as BspC to interact with vimentin expressed by the endothelial cells that form the blood-brain barrier (BBB). The BspC–vimentin interaction promotes GBS adherence to the BBB and facilitates the pathogenesis of GBS meningitis. Here we identified the vimentin-binding pocket of BspC within the variable (V-) domain and showed that an intact and accessible vimentin-binding pocket was required for GBS virulence. We used a virtual structure-based screen to identify compounds that could block the V-domain pocket and confirmed that chemically targeting the pocket could limit GBS adherence to the BBB as well as entry into the brain using our murine model of meningitis. Collectively, these results lay the foundation for development of novel anti-virulence therapies to prevent GBS pathogenesis.

## Introduction

Antibiotic treatment has historically been the primary method for combating potentially deadly bacterial diseases since the first release of Penicillin in 1941; unfortunately, the first penicillin-resistant bacterial strain was identified only one year later in 1942 [1]. The rapid development of antibiotic resistance in bacteria has been a constant and increasing trend to the point where experts believe that we are at the edge of a global medical crisis. The rise of increasing antibiotic resistance can be explained by the fact that most antibiotics provide a selective pressure for development of resistance [1–4]. Both bacteriostatic and bactericidal antibiotics aim to prevent growth and kill the target bacteria, while an individual bacterium that develops resistance to antibiotics is able to proliferate despite the presence of the antibiotic. Additionally, broad-spectrum antibiotics typically damage the commensal and often beneficial organisms within the microbiota, leading to further complications [5]. Still, due to the high rates of morbidity and mortality associated with bacterial infections a method to limit pathogenesis is required. One alternative to antibiotic therapy is the use of “anti-virulence drugs” which block bacterial virulence factors to prevent disease. Blocking of virulence factors such as adhesins, toxins, and secretion systems can limit pathogenesis without impacting bacterial viability, thereby avoiding a selective pressure for development of resistance [2–4]. Furthermore, the specificity of anti-virulence drugs prevents significant off-target effects on commensal microbes.

We recently identified a virulence factor called BspC in *Streptococcus agalactiae*, otherwise known as Group B *Streptococcus* (GBS) [6], and aim to use this as a model for systematic characterization of anti-virulence drug targeting during GBS infection. GBS asymptomatically colonizes approximately one third of women, and up to 70% of infants born to mothers with GBS will also become colonized [7,8]. Of those infants, 1–2% will develop invasive disease; currently GBS is a leading cause of neonatal bacterial meningitis worldwide [8,9]. Bacterial meningitis, a major cause of child mortality, is the severe inflammation of the meninges in response to infection following bacterial transit across endothelial barriers such as the blood-brain barrier (BBB) [10,11]. Prior to causing bacterial meningitis GBS must first interact with the endothelial cells comprising the BBB. Multiple GBS surface-associated factors have been shown to contribute to the initial interaction including: lipoteichoic acid (LTA) [12], pili [13], serine-rich repeat (Srr) proteins [14,15], streptococcal fibronectin-binding protein (SfbA) [16], and most recently the Group B streptococcal surface protein C (BspC) [6]. While many of these have been shown to interact with extracellular matrix components, BspC is the first GBS surface adhesin shown to interact directly with an endothelial cell receptor. BspC binds vimentin, a type III intermediate filament protein that is highly expressed in many tissues including brain endothelial cells [17,18]. The BspC-vimentin interaction is required for development of meningitis; therefore, this interaction represents an ideal candidate to be targeted for anti-virulence therapy. While BspC is known to interact with the C-terminus of vimentin, the specific portion of BspC that facilitates this interaction is unknown and requires identification prior to drug screening [6].

BspC belongs to a family of multifunctional proteins known as Antigen type I/II (AgI/II) proteins that are widely distributed among streptococcal species and were first identified to be encoded on integrative and conjugative elements in GBS [19]. AgI/II proteins exhibit a range of functions including promoting colonization of various tissues and contributing to diseases including dental caries, pneumonia, endocarditis, and meningitis [20,21]. The structure of AgI/II proteins is highly conserved and consists of a stalk made by the interaction between alanine rich (A) and proline rich (P) domains, which is stabilized by an interaction between the N-terminal (N) and globular C-terminal (C) domains [21]. Collectively, this stalk like structure projects a globular variable (V) domain away from the cell surface. The V-domain of AgI/II proteins often contains a lectin-like cleft or pocket that has been implicated in various functions, including ligand binding [21].Within GBS there are four AgI/II homologs designated BspA-D [22]. BspA and BspB share 90% sequence identity. BspC and BspD both lack two sequences that are present in BspA/B, one consisting of ~51 amino acids in the A domain and the other consisting of ~25 amino acids in the P domain. BspC and BspD share >99% identity with the main difference being that BspD lacks a leader peptide required for Sec dependent translocation to the surface. Although the V-domain was designated as such because of variability in this region when comparing AgI/II proteins from different species, the majority of sequence differences between BspA-D occur in the A and P domains, while the V-domain displays 96–100% sequence similarity in GBS [21,22] **(S1A and S1B Fig)**.

The structure of the BspA V-domain was recently elucidated via x-ray crystallography and was shown to be important for binding glycoprotein-340 (gp-340), a common receptor for AgI/II proteins [22]. While other domains of AgI/II proteins have been similarly shown to interact with various receptors [21], based on the Bsp sequence conservation and receptor binding ability of the BspA V-domain we hypothesized that that V-domain of BspC would contribute to interaction with its host receptor, vimentin. Here we broadly characterize the vimentin-binding pocket of the BspC V-domain. We then used the high-throughput power of a virtual structure-based screen to identify existing FDA approved drugs that can specifically block the vimentin-binding pocket and its role in the pathogenesis of GBS meningitis.

## Results

### Bsp distribution and conservation

**Fig 1:**
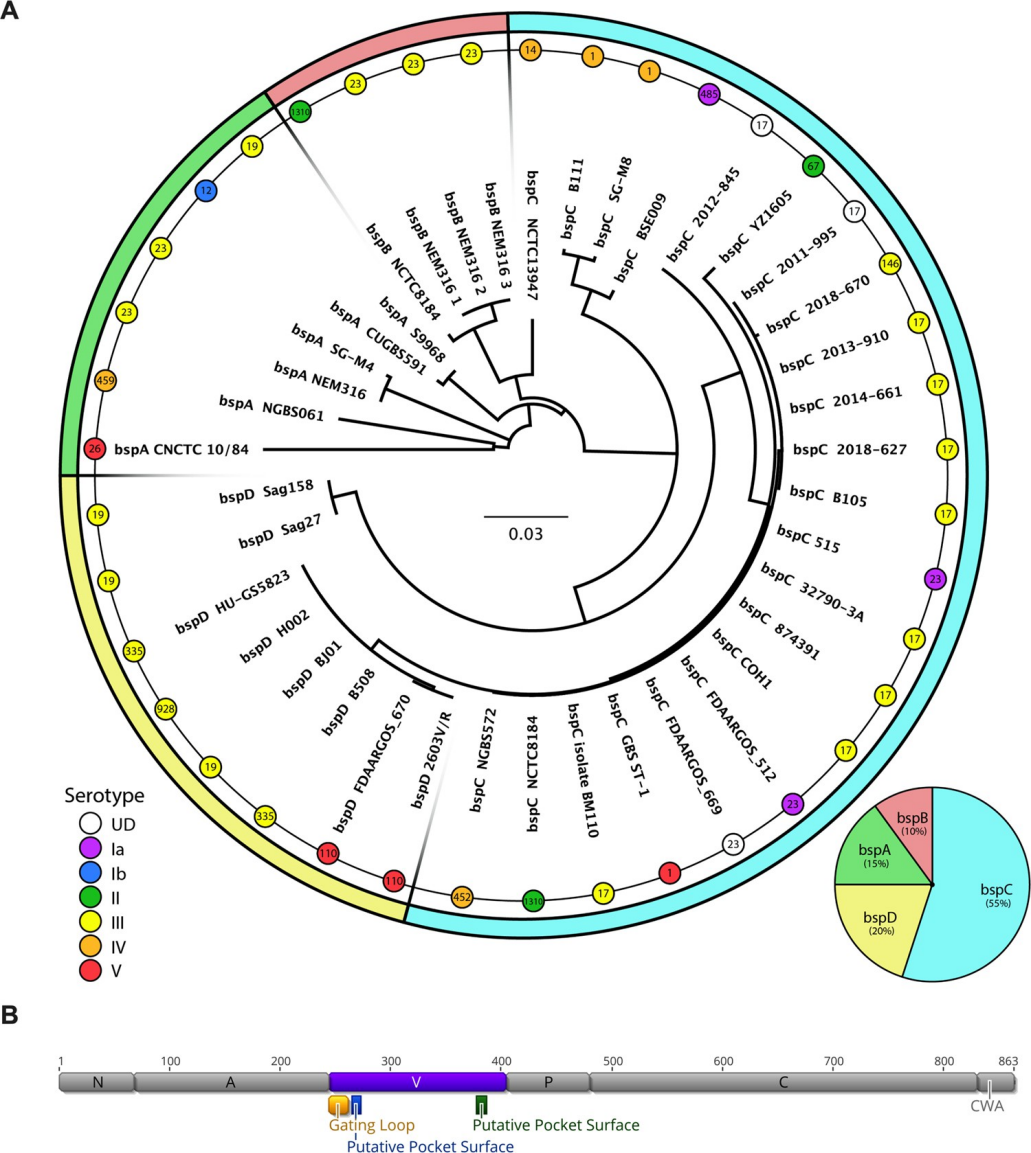
Bsp Distribution and Conservation. **(A)** The amino acid sequences listed in S1B Fig were used to form a neighbor-joining phylogenetic tree calculated by a Blosum80 cost matrix and Jukes-Cantor genetic distance model. The scale bar in the center indicates the distance representing 0.03 substitutions per site. Serotype of each strain is indicated by a colored node, while sequence type is indicated by a number within each node. This tree is overlaid on a pie chart which shows the distribution of each *bsp* homolog (also shown in the bottom right). **(B)** The gene structure for COH1 *bspC* is shown, with specific domains and relevant regions within the V-domain annotated.

Rego *et al*. last examined the distribution of *bspA*-*D* in 2016 by using the completed GBS genome sequences that were available on NCBI at that time [22]. As there have been many new GBS genomes added/completed since then, we took a similar approach to update our understanding of *bsp* distribution within GBS. An alignment of the amino acid sequences for all these *bsp* homologs displays the genetic differences shown in **S1B Fig**. A phylogenetic tree of all *bsp* genes and the ratio for each homolog is shown in **Fig 1A.** Of the 135 currently available completed GBS genomes, 36 possess a *bsp* homolog. This includes multiple well studied GBS clinical isolates such as COH1, 515, NEM316, and 2603V/R. Interestingly, BspC accounted for 55% of the homologs overall.

We also sought to determine the distribution of *bsp* homologs with relation to capsular serotype and sequence type, as these factors can correlate with disease. In particular, the serotype III sequence type (ST) 17 strains are known to be hypervirulent and are overrepresented in the strains that cause meningitis [23,24]. A Fisher’s Exact test was used to determine that while the distribution of *bsp* homologs with relation to capsular serotype was not significant (p = 0.4673), the *bsp* distribution was dependent on sequence type (p = 0.00009) (**Fig 1A**). Interestingly, we observed that in all instances where a ST-17 strain possessed a *bsp* gene, it was always *bspC*. Due to the known role of BspC in the pathogenesis of GBS meningitis, this distribution pattern may indicate a contribution of BspC to the hypervirulence associated with serotype III ST-17 strains.

As we hypothesized that the V-domain was important for BspC interaction with vimentin and would serve as an ideal anti-virulence drug target, we sought to determine the level of conservation of the BspC V-domain. An alignment of the V-domain from the available *bspC* genes shows that the V-domain of BspC appears to be highly conserved, with 98% amino acid identity (**S1B and S1C Fig**). The genetic domain organization of the COH1 BspC, as well as important regions within the V-domain such as a gating loop and regions predicted to form the surface of a pocket are shown in **Fig 1B.**

### The BspC V-domain binds vimentin and contributes to GBS adherence

**Fig 2:**
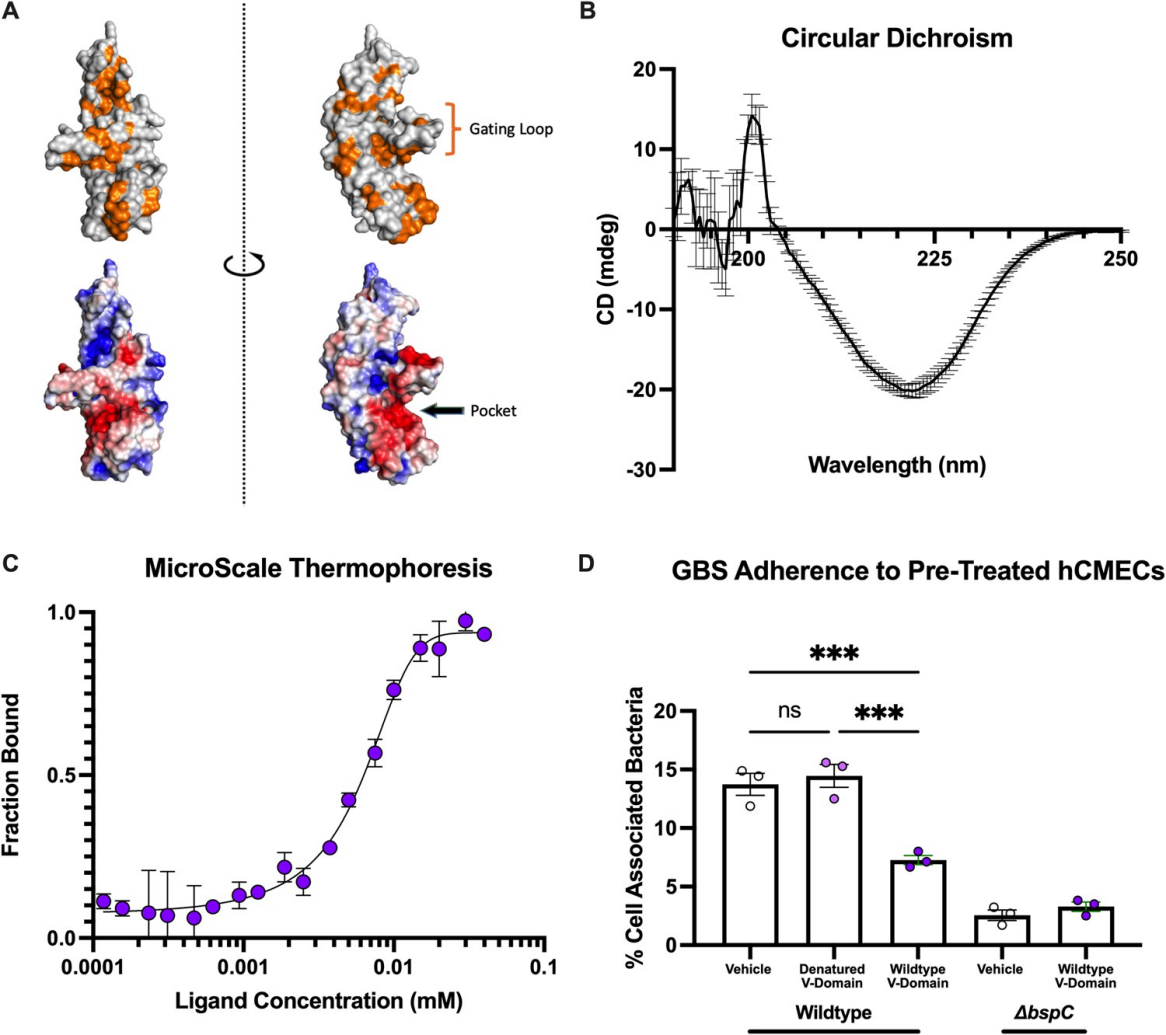
Mapping vimentin binding activity to the BspC V-domain. **(A)** The V-domain of BspC was modeled using BspA as a template. Surface residues are shown with hydrophobic residues colored in orange (top) and electrostatic potential as calculated by APBS (bottom) colored with red indicating higher potential and blue indicating lower potential. APBS calculation and visualization were performed using PyMOL. **(B)** Circular dichroism spectrum of a 1 mg/mL solution of the BspC V-domain. **(C)** The V-domain was added to 20 nM vimentin at the indicated concentrations prior to measuring the microscale thermophoresis dose response curve quantifying the dissociation constant. **(D)** hCMECs were pretreated with PBS (vehicle), the V-domain added to a concentration of 10 μM, or 10 μM of denatured V-domain 30 minutes prior to infection. CFU were plated and to assess V-domain blocking of GBS adherence after 30 minutes of incubation. B-D display means from 3 independent experiments. Error bars indicate standard error of the mean. Statistical analysis: (D) One-way ANOVA with Tukey’s multiple comparisons. ***, P < 0.0005.

As the V-domain is highly conserved across all *bsp* subtypes, we used the BspC V-domain sequence from the hypervirulent ST-17 meningitis-associated clinical isolate COH1 for homology based structural modeling using SWISSMODEL [25] with the BspA V-domain crystal structure [22] as a template. Visualization of the model revealed a hydrophobic pocket with high electrostatic potential guarded by a putative gating loop **(Fig 2A)**, representing a potential binding pocket. To confirm whether the V-domain was sufficient for interaction with vimentin, we cloned and purified a His6 tagged V-domain protein and validated it using circular dichroism (CD). The CD spectrum of the purified BspC V-domain showed a strong negative CD peak at 220 nm indicative of mostly beta sheets **(Fig 2B)**, which corresponds to the known structure of the BspA V-domain [22,26]. Direct binding of this purified V-domain protein to vimentin was assessed using microscale thermophoresis (MST), a biophysical technique that can be used to measure protein-protein interaction strength based on the response of a fluorescently labeled protein to increasing concentrations of another protein. Full-length BspC and vimentin were previously investigated using MST and were shown to interact with an estimated dissociation constant (*K*_d_) of 3.39μM [6]. Our MST results indicate that the BspC V-domain is sufficient for interaction with vimentin with the comparable estimated *K*_d_ of 7.14 μM **(Fig 2C)**.

It was previously observed that GBS Δ*bspC* displays decreased adherence to human cerebral microvascular endothelial cells (hCMECs), an *in vitro* model for the BBB [6]. We investigated the ability of purified V-domain protein to competitively inhibit GBS adherence in a BspC dependent manner by treating hCMEC monolayers with the protein prior to infection **(Fig 2D)**. Pre-treatment with the BspC V-domain significantly decreased WT GBS adherence to the hCMECs but had no impact on adherence by the Δ*bspC* mutant. A control pre-treatment with a heat denatured V-domain protein was unable to block WT GBS adherence.

### Flexibility of the V-domain gating loop is required for vimentin binding and GBS adherence

**Fig 3:**
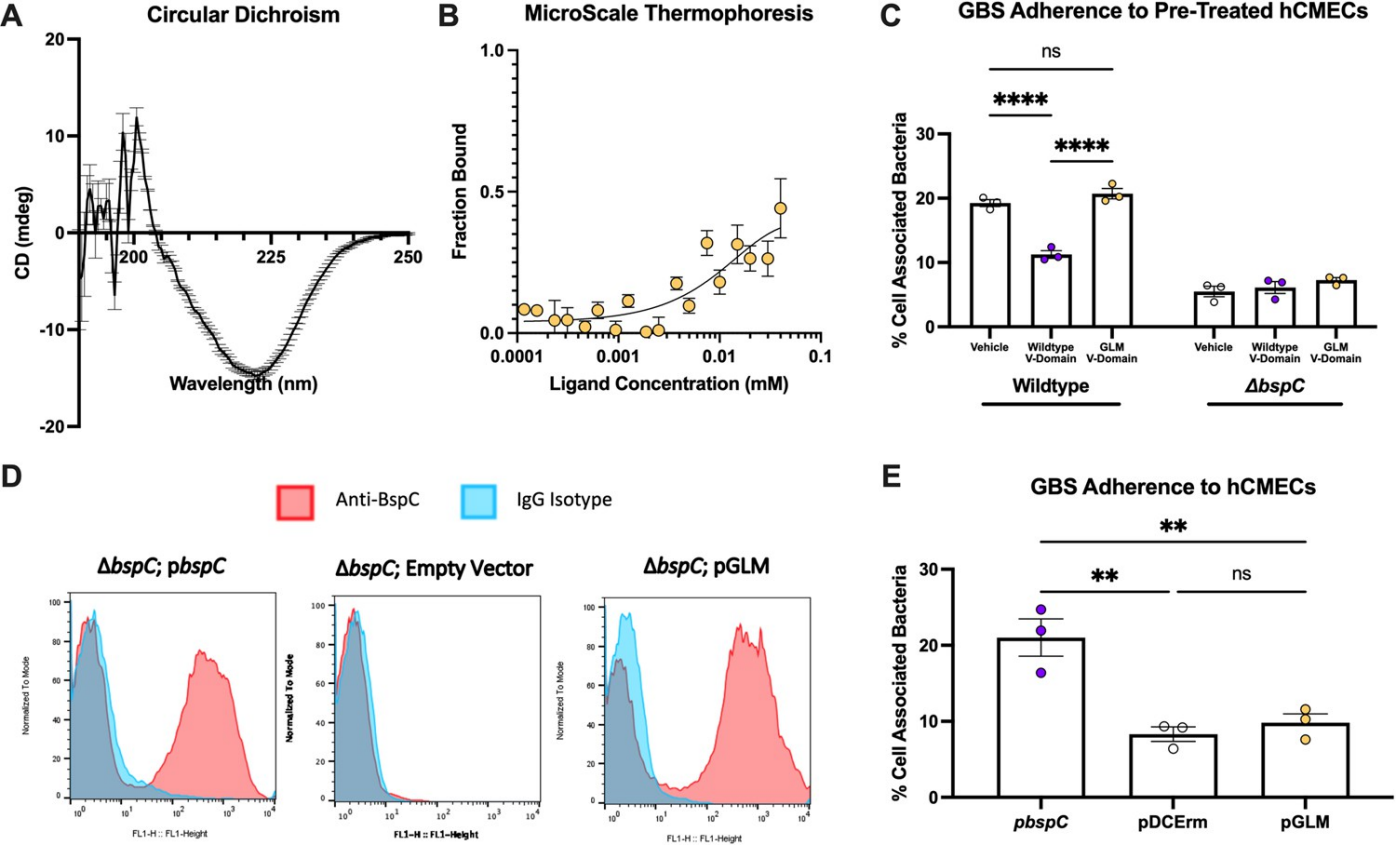
Characterization of the role of the V-domain gating loop. **(A)** Circular dichroism spectrum of a 1 mg/mL solution of the BspC GLM V-domain. **(B)** The GLM V-domain was added to 20 nM vimentin at the indicated concentrations prior to measuring the microscale thermophoresis dose response curve quantifying the dissociation constant. **(C)** hCMECs were pretreated with PBS (vehicle), the V-domain added to a concentration of 10 μM, or 10 μM of the GLM V-domain 30 minutes prior to infection. CFU were plated to assess V-domain blocking of GBS adherence after 30 minutes of incubation. **(D)** COH1*ΔbspC* GBS containing pDCErm*bspC* (p*bspC*), pDCErm*bspC* GLM (pGLM), or the empty vector (pDCErm) were stained with an anti-BspC antibody or isotype control without permeabilization. Surface bound antibody was measured via flow cytometry. **(E)** The same strains used in **(D)** were used to infect a monolayer of hCMECs. CFU were plated to assess adherence after 30 minutes of incubation. A-C and E display means pooled data from 3 independent experiments. D displays representative histograms taken from one of three independent flow experiments. Error bars indicate standard error of the mean. Statistical analysis: Two-way ANOVA with Tukey’s multiple comparisons (C) and One-way ANOVA with Tukey’s multiple comparisons (E). **, P < 0.005; ****, P < 0.00005.

Next, we sought to confirm that the hydrophobic pocket with high electrostatic potential, shown in **Fig 2A**, was responsible for the V-domain’s ability to bind vimentin. The pocket is guarded by a putative gating loop that has flexible alanine hinges on either side, potentially blocking or stabilizing interactions within the pocket under various conditions. Alanine to proline substitutions have often been used to increase the rigidity of certain regions to investigate function [27–29]. To limit the flexibility of the gating loop and potentially block access to the pocket itself, we used site directed mutagenesis to convert alanine residues 250 and 259 into proline residues. This A250P/A259P mutant protein was termed the Gating Loop Mutant (GLM). The V-domain of the GLM was purified and investigated in a similar manner to the WT V-domain protein. The CD spectrum of the GLM V-domain protein still displayed a negative peak at 220 nm corresponding to a secondary structure of mostly beta sheets **(Fig 3A)**, as was observed with the WT V-domain protein. MST analysis, however, revealed a decrease in interaction of the GLM V-domain protein and vimentin with an estimated *K*_d_ of 41.9 μM **(Fig 3B)**. The decreased interaction of the GLM V-domain with vimentin was significant enough to abolish the ability to block WT GBS binding to hCMEC **(Fig 3C)**.

To investigate the impact of gating loop mutations within the context of the full-length protein expressed on the bacterial surface, we cloned the full-length WT *bspC* and full-length *bspC* containing the GLM sequence into the pDCErm expression vector (pGLM) and transformed it into the COH1 Δ*bspC* background. Bacterial growth was not impacted by expression of these proteins **(S2A Fig)**. To confirm that this mutant protein was being expressed on the GBS surface, we used flow cytometry with immunofluorescent staining using BspC specific polyclonal antibodies. The pGLM and WT BspC complementation (p*bspC*) strains both displayed a shift in fluorescence as compared to the IgG isotype control which was not observed with the empty vector control **(Fig 3D)**. While we observed that a subset of cells measurably expressed BspC proteins, there was no difference in the percent of cells that were positive for GLM or WT BspC **(S2B Fig)**. We then measured adherence of these strains to hCMECs and observed that the GLM was unable to complement the Δ*bspC* associated adherence defect **(Fig 3E)**. Together, these results indicate that flexibility of the gating loop is required for BspC interaction with vimentin and BspC dependent adherence of GBS to hCMECs.

### An accessible V-domain binding pocket is required for the pathogenesis of GBS meningitis

**Fig 4:**
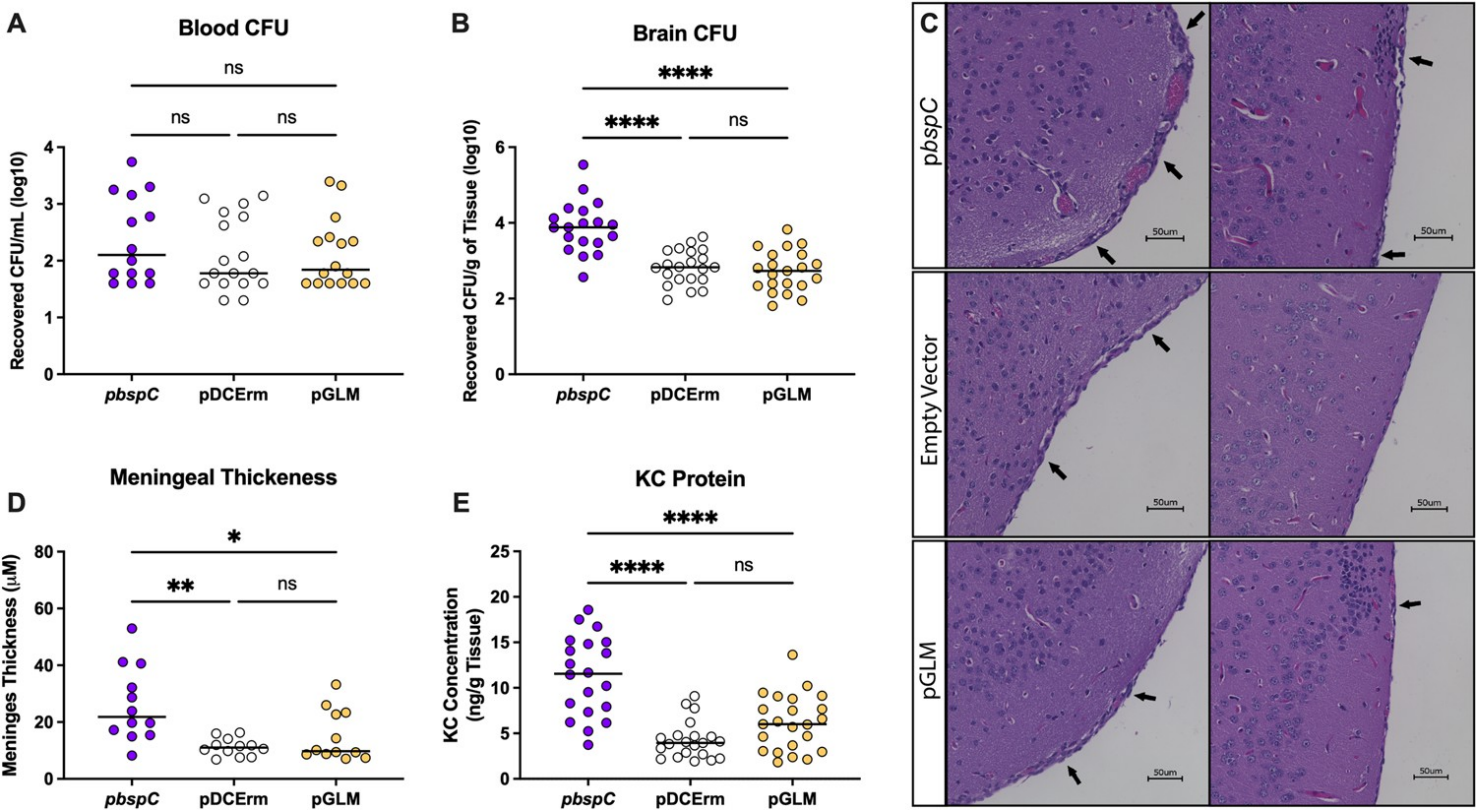
Flexibility of the Pocket-Guarding Gating Loop is Required for GBS Meningitis. Mice were infected with ~4x10^8^ CFU of COH1*ΔbspC* GBS containing pDCErm*bspC* (p*bspC*), the empty vector (pDCErm), or pDCErm*bspC* GLM (pGLM) and then sacrificed after 48 hours. **(A)** Half of each mouse brain was homogenized and plated on THA-Erm to determine bacterial burden. **(B)** Blood from each mouse was also plated on THA-Erm to determine bacterial burden. For **(A)** and **(B)**, data is pooled from three independent experiments, where each dot represents an individual mouse, and lines indicate statistical means. **(C)** H&E stains showing representative images of the leptomeninges from two individual mice per group. Arrows indicate areas of leukocyte infiltration and meningeal thickening. **(D)** ImageJ was used to analyze meningeal thickness from 3 mice per group. Two images were taken of each brain, and three distinct regions per image were measured with each dot representing an individual region. **(E)** KC protein concentration was quantified via ELISA from the brain homogenates shown in A. Data for E is pooled from three independent experiments, where each dot represents an individual mouse brain, and lines indicate statistical means. Statistical analysis: (A, B, C, and D) One-way ANOVA with Tukey’s multiple comparisons. *, P < 0.05; **, P < 0.005; ****, P < 0.00005.

Our results thus far suggest a primary role for the V-domain and binding pocket accessibility in promoting BspC interaction with vimentin and brain endothelial cells. We hypothesized that this *in vitro* phenotype would translate into a diminished bacterial ability to penetrate the BBB and produce meningitis *in vivo*. Using our established *in vivo* murine model for hematogenous meningitis [6,12,30,31], we confirmed that vector complementation of Δ*bspC* restored infection to levels comparable to the WT COH1 infection **(S2C and S2D Fig)**. We then infected groups of mice with COH1 Δ*bspC* containing the WT *bspC* complementation vector (p*bspC*), the empty vector (pDCErm), or the *bspC* GLM complementation vector (pGLM). After 48 hours mice were euthanized and brain and blood were harvested to enumerate bacterial load (colony forming units, CFUs). The plating was done on THA and on THA supplemented with erythromycin to confirm that there was no plasmid loss in any of the strains during the experimental time frame **(S2E and S2F Fig)**. We observed no significant difference in bacteria recovered from the blood between all strains **(Fig 4A)**. Despite the comparable levels of bacteremia, there was a significant reduction of GBS expressing pGLM in the brain compared to the level of the BspC expressing strain **(Fig 4B)** indicating that the GLM expressing strain had decreased ability to enter the brain.

Brain sections were also stained with hematoxylin and eosin and were investigated for the meningeal thickening that is a hallmark of meningitis **(Fig 4C)**. While some leukocyte infiltration and meningeal thickening was observed across all groups, the mice infected with the WT BspC expressing strain displayed the most dramatic increases. The meningeal thickness was also measured and quantified using ImageJ [32] to confirm that the meninges of mice infected with the WT BspC expressing strain were on average thicker than those of mice infected with either the empty vector or GLM expressing strains **(Fig 4D)**. Finally, an ELISA measuring the concentration of neutrophil chemoattractant KC protein in brain homogenates demonstrated that infection with the GLM expressing strain resulted in less KC abundance compared to the WT BspC expressing strain **(Fig 4E)**. Taken together these results indicate that an accessible V-domain pocket is required for the BspC dependent development of GBS meningitis.

### Disruption of the V-domain pocket decreases vimentin binding, GBS adherence, and meningitis

**Fig 5:**
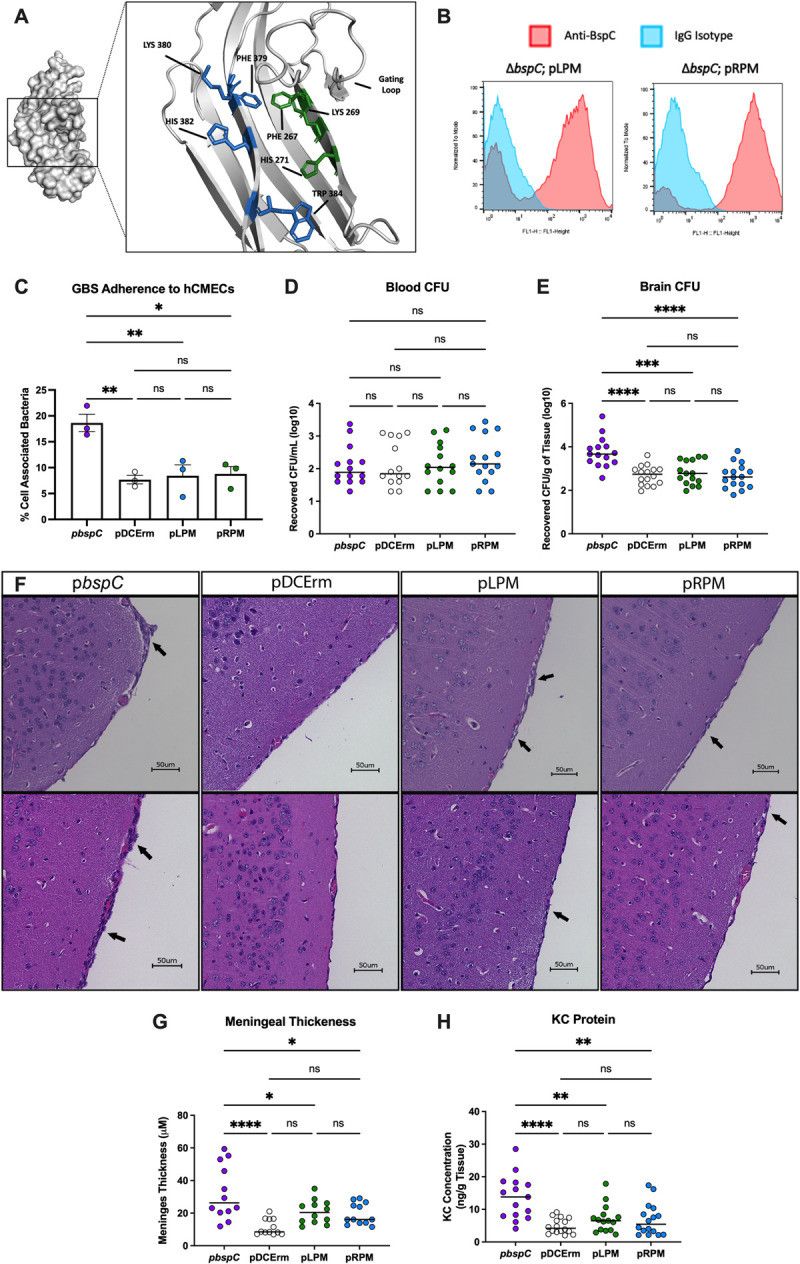
**The V-domain pocket contributes to BspC Function (A)** Zoomed in view of the V-domain pocket with residues targeted for mutagenesis. Residues shown in blue were targeted to construct LPM, and residues shown in green were targeted to construct RPM. Visualization done using PyMOL. **(B)** COH1 *ΔbspC* GBS containing pDCErm*bspC* (p*bspC*), pDCErm*bspC* LPM (pLPM), pDCErm*bspC* RPM (pRPM) or the empty vector (pDCErm) were stained with an anti-BspC antibody or isotype control without permeabilization. Surface bound antibody was measured via flow cytometry. **(C)** The same strains used in **(B)** were used to infect a monolayer of hCMECs. CFU were plated to assess adherence after 30 minutes of incubation. **(D-H)** Mice were infected with ~4x10^8^ CFU of the same strains used in **(B)** and then sacrificed after 48 hours. **(D)** Half of each mouse brain was homogenized and plated on THA-Erm to determine bacterial burden. **(E)** Blood from each mouse was also plated on THA-Erm to determine bacterial burden. For **(D)** and **(E)**, data is pooled from two independent experiments, each dot represents an individual mouse, and lines indicate statistical means. **(F)** H&E stains showing the leptomeninges from two individual mice per group, one from each independent experiment. Arrows indicate areas of leukocyte infiltration and meningeal thickening. **(G)** ImageJ was used to analyze meningeal thickness from 5 mice per group from the two independent experiments. Two images were taken of each brain, and three points per image were measured with each dot representing an individual point. **(H)** KC protein concentration was quantified via ELISA from brain homogenates shown in D. Data for H is pooled from two independent experiments, where each dot represents an individual mouse brain, and lines indicate statistical means. Statistical analysis: (C, D, E, G, and H) One-way ANOVA with Tukey’s multiple comparisons. *, P < 0.05; **, P < 0.005; ***, P < 0.0005; ****, P < 0.00005.

As our data demonstrate the importance of the gating loop within the BspC V-domain, we sought to confirm that the binding pocket itself was important for BspC V-domain function. The surface of the pocket is composed of the edge of two anti-parallel beta-sheets that contain multiple residues highly conserved across homologs. We targeted both the left and right side of the pocket surface using site-directed mutagenesis to create two multi-point mutants. Mutations within the left portion of the pocket (F267A, F269A, H271A) resulted in the mutant termed the Left Pocket Mutant (LPM), while mutations within the right portion of the pocket (F379A, K380E, H382A, and W384A) resulted in the mutant termed the Right Pocket Mutant (RPM) **(Fig 5A).** The LPM mutations were chosen to remove the hydrogen donor/acceptors phenylalanine and histidine to decrease the number of potential ligand interaction points while also slightly neutralizing the charge by removing the histidine. The RPM mutations were similarly chosen to remove the hydrogen donor/acceptor phenylalanine and hydrogen donors tryptophan and lysine, while probing the impact of converting the positively charged lysine into a negatively charged glutamic acid residue. Surface expression of LPM and RPM on GBS within the COH1 Δ*bspC* background was confirmed by flow cytometry as described above **(Fig 5B)**. Bacterial growth was unaffected by expression of these mutant proteins and there was no difference in the percent of cells expressing the LPM and RPM mutants compared to WT *bspC* **(S2A and S2B Fig)**.

The GBS LPM and RPM expressing strains displayed a decreased ability to complement the Δ*bspC* associated defect in adherence to hCMECs (**Fig 5C)**, thus we also examined the ability of these strains to cause meningitis *in vivo*. As described above, mice were infected with either the WT BspC expressing strain (p*bspC*), the empty vector control (pDCErm), the LPM (pLMP) or the RPM (pRPM) expressing strains. After 48 hours mice were sacrificed, and their brains and blood were harvested and plated for CFU enumeration. The plating was done on THA and on THA supplemented with erythromycin to confirm that there was no significant loss of the plasmids in any of the strains **(S2G and S2H Fig)**. As observed with the GLM expressing strain, both LPM and RPM expressing strains displayed equal levels of bacteremia as control strains **(Fig 5D)**, but had a significant decrease in their ability to enter the brain **(Fig 5E)**. Meninges observed after brain sectioning and H&E staining also displayed a significant reduction in thickening in mice infected with both LPM and RPM expressing strains compared to the WT BspC expressing strain **(Fig 5F and 5G)**. Finally, an ELISA measuring the concentration of KC protein in brain homogenates revealed that the LPM and RPM expressing strains caused a reduced amount of neutrophil chemoattractant signaling as compared to the WT BspC expressing strain **(Fig 5H)**. These results demonstrate the importance of the pocket contained within the BspC V-domain for GBS interaction with the BBB and development of meningitis *in vivo*.

### Drugs targeting the V-domain binding pocket can block interaction with vimentin

We next hypothesized that vimentin-binding pocket in the V-domain of BspC would be an ideal site to target therapeutically. We used a structure based virtual screening approach, utilizing the docking program PLANTS to screen the e-Drug3D library of FDA-approved drugs against the pocket contained in the WT BspC V-domain [33,34] (see Materials and Methods for details). The top 10 hits from this screen are shown in **Table 1.** Of these drugs, Cobicistat, Carfilzomib, Tafluprost, Venetoclax, Paliperidone Palmitate, and Lapatinib were chosen for further investigation as they were readily commercially available. Deferoxamine was intentionally excluded as its known iron chelating properties would likely affect bacterial growth and confound results [35].

**Table 1:** Top 10 hits from the PLANTS virtual structure-based screen of e-Drug3D library of FDA approved drugs against the GBS BspC V-domain.

Rank	Score (%)	Name	MW
1	94.87	Glycerol Phenylbutyrate	530.63
2	94.20	Cobicistat	776.03
3	93.87	Carfilzomib	719.91
4	92.91	Latanoprostene Bunod	507.61
5	92.34	Tafluprost	409.44
6	92.29	Venetoclax	868.44
7	92.11	Paliperidone Palmitate	664.89
8	91.94	Deferoxamine	560.69
9	91.71	Lapatinib	581.06
10	91.54	Fluphenazine Decanoate	591.77

**Fig 6:**
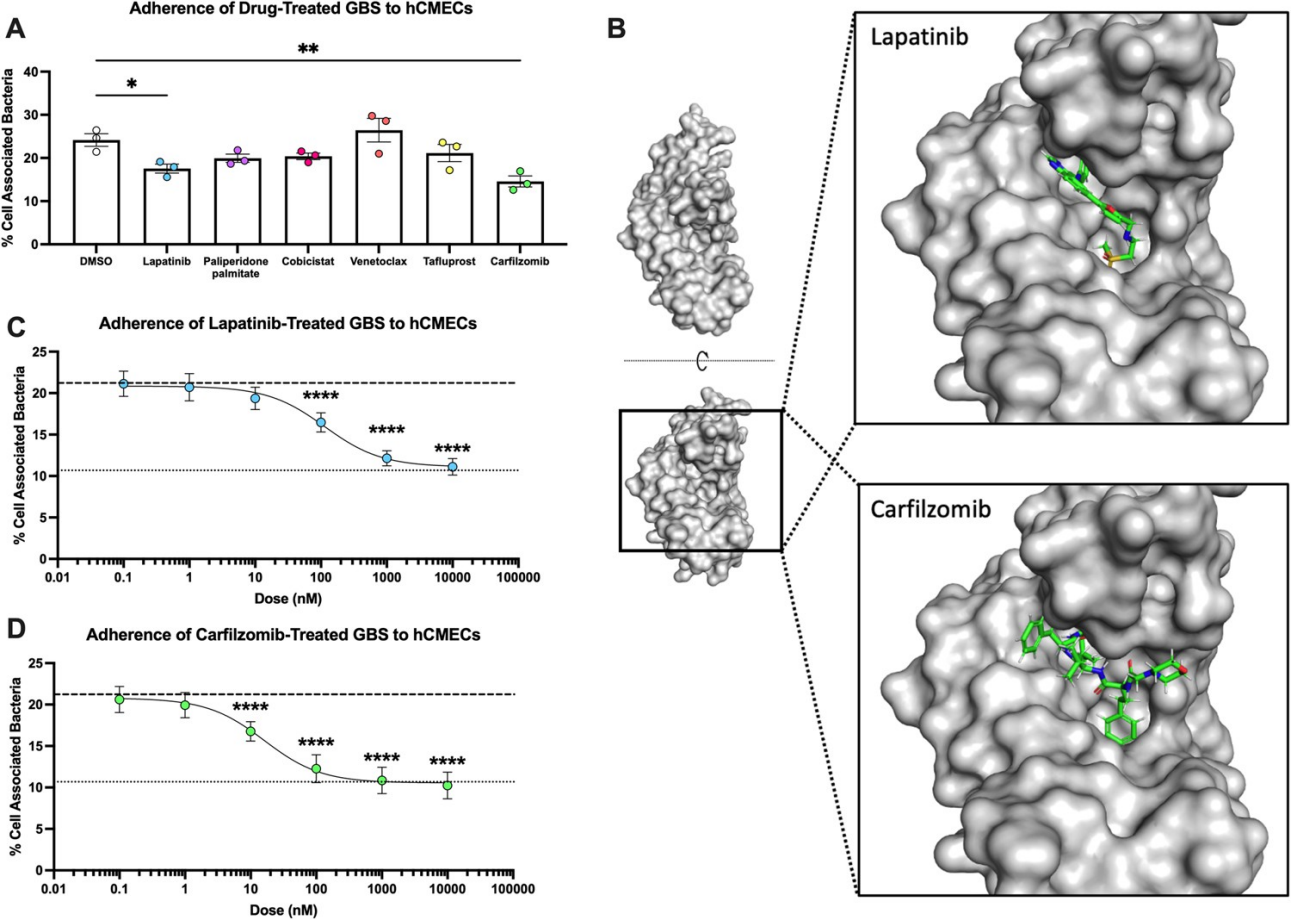
**Lapatinib and Carfilzomib bind the BspC V-domain and prevent adherence to hCMECs (A)** GBS was pretreated with DMSO (vehicle), 10 μM of Lapatinib, Cobicistat, Venetoclax, or Carfilzomib, or 1 μM of Paliperidone Palmitate or Tafluprost 30 minutes prior to infection of hCMECs. CFU were plated to assess blocking of GBS adherence after 30 minutes of incubation. **(B)** A model of Lapatinib (top) and Carfilzomib (bottom) bound to the V-domain pocket. Visualization done using PyMOL. **(C and D)** GBS was pretreated with either DMSO, Lapatinib **(C)**, or Carfilzomib **(D)** at the indicated concentrations 30 minutes prior to infection of hCMECs. CFU were plated to assess blocking of GBS adherence after 30 minutes of incubation. The dashed line indicates the mean adherence of WT GBS pretreated with DMSO, while the dotted line indicates the mean adherence of the *ΔbspC* mutant pretreated with DMSO or the drugs. Pooled data from three independent experiments is shown. Error bars indicate standard error of the mean. Statistical analysis: (A) One-way ANOVA with Tukey’s multiple comparisons. *, P < 0.05; **, P < 0.005; ****, P < 0.00005.

With the remaining drugs, we sought to identify a concentration that would not impair GBS growth **(S3A Fig).** We saw slight growth inhibition at 10 μM concentrations with Paliperidone Palmitate and Tafluprost, but not at 1 μM. We also saw no inhibition with 10 μM concentrations of any of the other drugs; therefore, we chose to use 1 μM treatment with Paliperidone Palmitate and Tafluprost and 10 μM for the other compounds. In order to test the ability of these drugs to block BspC dependent GBS adherence, we incubated GBS WT and Δ*bspC* mutant strains with each drug for 30 minutes prior to infecting an hCMEC monolayer. This screen revealed that pretreatments by both Lapatinib and Carfilzomib significantly reduced GBS adherence to hCMECs **(Fig 6A)**, while there was no observed reduction in adherence of the Δ*bspC* strain **(S3B Fig)**. Of note, adherence of WT GBS pretreated with Carfilzomib was reduced to the level of the Δ*bspC* mutant treated with the vehicle control. The predicted binding of Lapatinib and Carfilzomib to the V-domain pocket that was generated by the PLANTS docking program is shown in **Fig 6B.** Both drugs can be seen bound within the hydrophobic region of the V-domain pocket **(Fig 2A)**, seemingly stabilized by the gating loop.

Due to the efficacy of both Lapatinib and Carfilzomib in blocking GBS-hCMEC interaction, we decided to further characterize these two drugs. We saw no impact on hCMEC survival as compared to the vehicle control when treating the cells with only Lapatinib or Carfilzomib at the final drug concentrations that cells were exposed to during the adherence assays **(S3C Fig)**. Treating the hCMECs with Lapatinib or Carfilzomib prior to bacterial infection also did not affect GBS adherence **(S3D Fig),** suggesting that the drugs are not changing the endothelial cells in a way that then impacts bacterial attachment. Finally, we measured the ability of both Lapatinib and Carfilzomib to block GBS adherence with doses ranging from 0.1 nM—10 μM in order to estimate an IC50 for each. The IC50 for *bspC* dependent blocking of adherence was estimated to be 113.3 nM for Lapatinib, and 16.1 nM for Carfilzomib **(Fig 6C and 6D)**. Pretreatment of the Δ*bspC* mutant at the same doses had no effect on adherence **(S3E Fig).** Furthermore, we examined other GBS strains and determined that carfilzomib pretreatment blocked hCMEC adherence by GBS strains 515 (possesses *bspC*) and NEM316 (possesses *bspA* and *bspB*); but had no impact on adherence of CJB111 which lacks a *bsp* homolog **(S4 Fig)**. These data indicate that chemically targeting the BspC V-domain pocket can block its function in directly promoting bacterial- host cell interactions.

**Fig 7:**
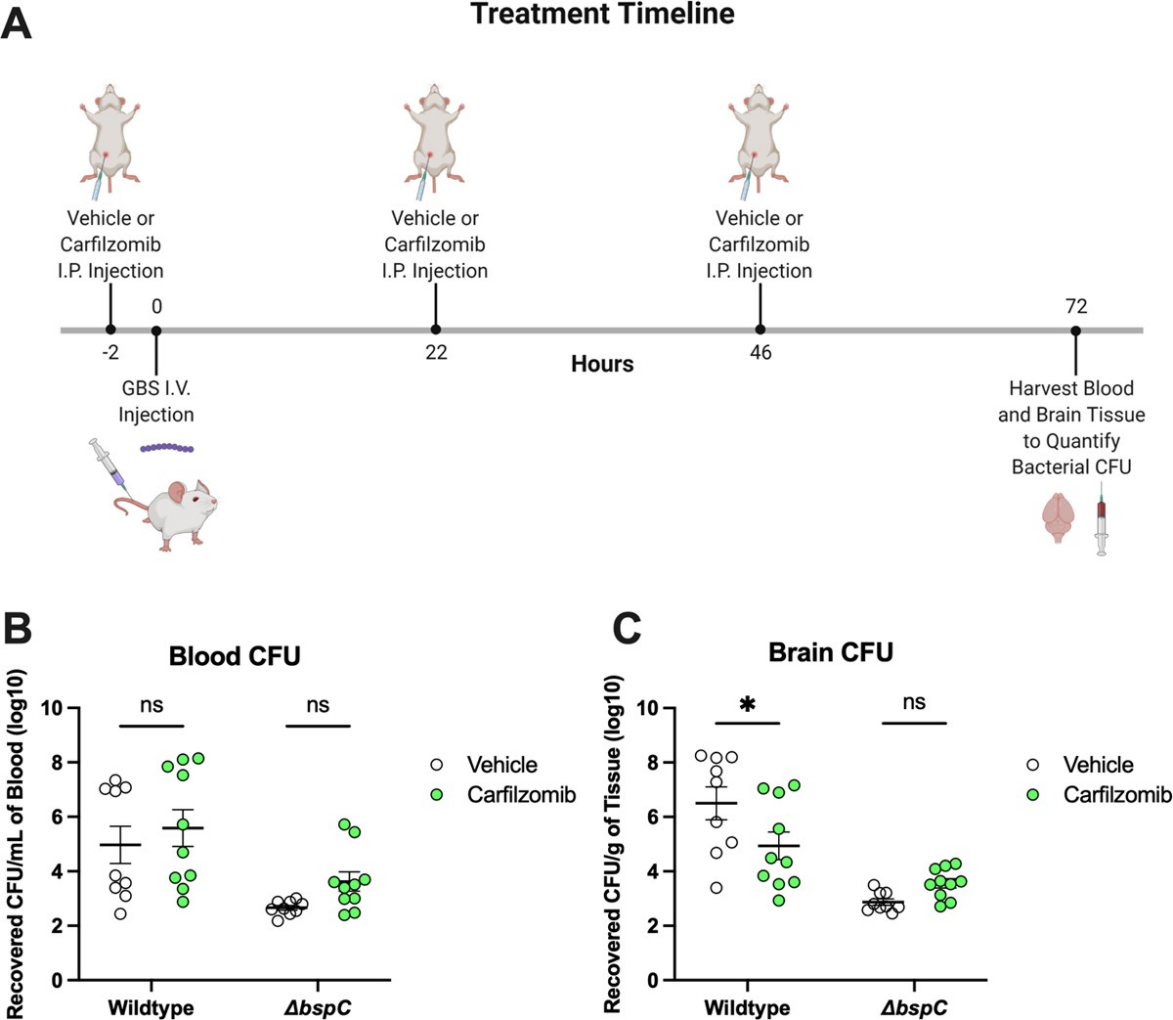
Carfilzomib Prevents GBS Brain Entry *in vivo*. **(A)** Schematic generated using BioRender displaying the experimental design and injection/infection timepoints for the comparison of Carfilzomib or vehicle (10% DMSO in sesame oil) treatments. Mice were infected with ~8.5x10^8^ CFU of either WT or Δ*bspC* GBS. Four WT GBS infected vehicle treated mice and three WT infected carfilzomib treated mice were euthanized early based disease severity. All remaining mice were euthanized at 72 hours post infection, at which time the brain tissues **(B)** and blood **(C)** were harvested for enumeration of bacterial CFU. Pooled data from two independent experiments is shown. Error bars indicate standard error of the mean. Statistical analysis: (A) Two-way ANOVA with Tukey’s multiple comparisons. *, P < 0.05.

Finally, we investigated the efficacy of blocking the BspC V-domain pocket for preventing disease *in vivo*. We chose to examine Carfilzomib due to its lower IC50. We administered intraperitoneal injections of 2.5 mg/kg Carfilzomib or the vehicle control every 24 hours during infection with either WT or Δ*bspC* mutant GBS until the mice were euthanized at 72 hours post infection, at which point brain tissue and blood were harvested to enumerate GBS CFU **(Fig 7A)**. Carfilzomib treated mice exhibited a significant reduction of bacterial penetration into the brain as compared to vehicle treated controls, with no significant differences in bacterial load observed within the bloodstream **(Fig 7B and 7C)**. These data suggest that therapeutically blocking the binding site within the BspC V-domain may be an effective strategy to block GBS entry into the brain.

## Discussion

**Fig 8:**
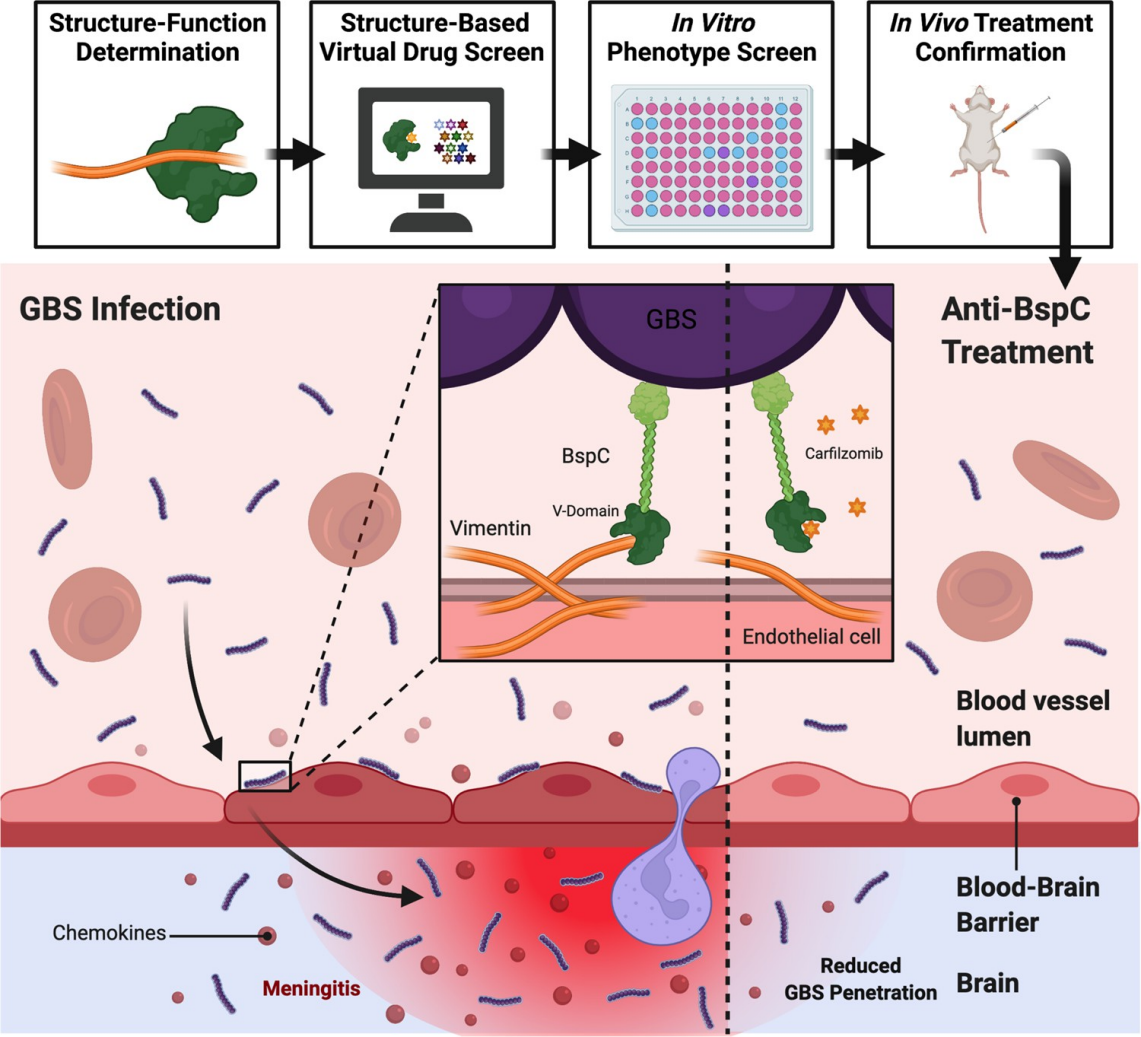
Summary of Anti-BspC Treatment to Reduce the Pathogenesis of GBS Meningitis. GBS utilizes BspC to interact with vimentin expressed by endothelial cells to adhere to the BBB endothelium and cause meningitis. Site-directed mutagenesis identified a vimentin-binding pocket contained within the BspC V-domain. This structure-function determination informed a virtual drug screen that yielded a list of drugs, two of which were confirmed to block BspC dependent adherence brain endothelial cells *in vitro*. Of these drugs, Carfilzomib was the most effective and was also able to prevent GBS entry into the brain *in vivo*. Figure generated using BioRender.

We recently demonstrated that GBS surface adhesin, BspC, interacts with host vimentin to initiate brain penetration and the development of meningitis [6]. Here we determine that the variable or V-domain of BspC is responsible for coordinating the interaction with vimentin and promoting GBS adherence to the BBB. Specifically, our data suggest that a gating loop–guarded pocket contained within the V-domain is the vimentin-binding region as mutations in either the gating loop or the pocket itself result in decreased GBS adherence to brain endothelium and entry into the brain. Furthermore, treatment with drugs predicted to fit within the pocket region of the BspC V-domain, such as lapatinib and carfilzomib, similarly decreased GBS attachment and brain penetration in a BspC dependent manner. Collectively these results indicate that the V-domain of BspC is responsible for coordinating the pathogenesis of GBS meningitis, and that this interaction could be chemically blocked to decrease disease severity **(Fig 8)**.

Targeting virulence factors has been used in many instances to prevent disease; in fact, FDA approved anti-virulence therapies already exist for treatment of *Clostridium botulinum*, *Bacillus anthracis* and *Clostridium difficile* by targeting toxins produced by each of these species [2]. Targeting of bacterial surface adhesins specifically has also been proven effective in many instances. Inhibition of the *Staphylococcus aureus* Sortase A (SrtA) prevents anchoring of many adhesins to the cell surface, resulting in attenuation of disease phenotypes [36,37]. Anti-virulence drugs that block specific adhesin binding or even adhesin production have also been developed to prevent infections by uropathogenic *Escherichia coli* (UPEC) [38–40], *Klebsiella pneumoniae* [41], and *Enterococcus faecalis* [42]. Similarly, the virtual structure-based screening approach has also been widely used to identify potential therapeutics that target specific proteins [4,33,43–47]. The program that we used for our virtual screen, PLANTS, itself has been cited in studies aiming to identify therapeutics for a variety of infections including targeting of DNA gyrase B in *E*. *coli* [48], the HIV-1 integrase strand transfer complex [49], dihydroorotase in *S*. *aureus* [50], and most recently there has been a significant effort to target the main protease (M^pro^) of SARS-CoV-2 [51–53].

Here we sought to utilize this process to target a specific GBS adhesin, BspC, that is present in hypervirulent strains associated with meningitis. The predicted structure of the BspC V-domain closely mimics that of the GBS BspA V-domain, which was expected due to the high level of protein sequence conservation between the V-domains of each homolog [22]. Furthermore, the circular dichroism experiments that were conducted on the purified BspC V-domain **(Fig 2B)** confirmed the largely beta-sheet dependent structure that was observed with the BspA V-domain. The BspA V-domain has been shown to bind the innate immune component known as glycoprotein-340 (gp-340), a known receptor for the *S*. *mutans* SpaP V-domain [22,54]. Gp-340 is highly expressed at mucosal surfaces such as within the oral cavity (the niche where interaction with SpaP takes place), lungs, or the vaginal tract; therefore, it is possible that BspC also serves to promote GBS pneumonia or colonization of the vaginal tract [55,56]. Additionally, vimentin expression is also known to be induced during the epithelial to mesenchymal transition (EMT), which occurs in the vaginal tract during GBS infection and has been shown to promote ascending infection into the uterus where it may cause further complications leading to still birth or pre-term labor [57–59]. While BspA and BspC have been shown to be sufficient for allowing bacterial adherence to vaginal epithelial cells [22,60], a more thorough investigation into the role of Bsp proteins in GBS colonization of the vaginal tract and ascending infection is needed. If Bsp dependent interactions within alternative niches such as the vaginal tract are also coordinated via the V-domain, then anti-virulence V-domain targeting therapies such as Carfilzomib could also be effective in preventing GBS disease in all niches where the Bsp V-domain contributes to virulence.

The pocket region and associated gating-loop were also predicted in the BspA V-domain. Our data indicate that both the pocket and the gating loop itself are important for binding of vimentin and attachment to brain endothelium. The gating loop may act to either secure interactions within the pocket, or to block the pocket and only allow interactions under certain conditions. The gating loop mutations that we chose to make were designed to remove putative flexibility from the region, thus it is possible that the mutations may have sealed off the pocket entirely or prevented any potential gating-loop dependent securing interactions from taking place. Although it is also possible that the bulky side chains of the proline residues directly blocked interactions within the pocket due to steric hinderance, or that the substitutions disrupted the V-domain folding in a manner undetectable by circular dichroism. Interestingly, the structure of the V-domain with this gating-loop associated pocket is not well conserved in the Antigen I/II (AgI/II) family of proteins in other streptococci. While the V-domain of AgI/II proteins is the most conserved region when comparing within a species, the term “variable domain” originates from the fact that the V-domain is the least conserved region when comparing across *Streptococcus* species [21]. For example, the V-domain of the *S*. *mutans* AgI/II protein SpaP has very different structure than that of the Bsp proteins, and completely lacks a gating loop region [54]. Interestingly despite these differences, when we performed the virtual structure-based screen using the SpaP V-domain as the target, we found that four of the top ten hits were shared between those from our initial screen, including Carfilzomib, which can be observed bound to SpaP (**S3F and S3G Fig)**. This indicates that Carfilzomib or analogous treatments may have a broader scope that could include diseases caused by other bacterial species that possess AgI/II homologs or structurally similar adhesins.

While each of the top ten hits shown in **Table 1** were similarly scored, we observed that only Lapatinib and Carfilzomib treatment reduced GBS adherence to hCMECs **(Fig 6A)**. Both drugs are FDA approved and are typically used as anti-cancer therapies, with Lapatinib targeting epidermal growth factor receptor (EGFR) and Carfilzomib targeting the proteasome. Studies regarding side effects during use in either pregnant women or newborns are incredibly limited. One study found no pregnancy or delivery complications in a group of women that were exposed to Lapatinib during pregnancy [61]. Phase I and II clinical trials of Lapatinib treatment in children for CNS malignancies both concluded that Lapatinib was well tolerated in children, however, the youngest patient between both studies was 1.2 years old so the impact on newborns has yet to be determined [62,63]. While Carfilzomib treatment is not recommended for pregnant women because embryo-fetal toxicity was observed in rabbits [64], another study that investigated Carfilzomib treatment of human children concluded that the treatment was well tolerated, although once again the youngest patient was one year old [65]. While we used a Carfilzomib dose comparable to that used in *in vivo* anti-cancer studies, it is a possibility that the threshold for allowable toxicity is higher when treating cancer than when treating infectious diseases; as such, drug optimization both in terms of design and dosage are important considerations before Carfilzomib, or an analogous drug, could be used to treat GBS infections. Additionally, the possibility that bacterial bound drug would disassociate and contribute to host cell cytotoxicity remains to be tested. Route of drug administration is similarly important; our studies used intraperitoneal injections as a proof of concept, but in humans Lapatinib is administered as an oral tablet, and Carfilzomib is administered as an intravenous injection[64,66]. The intravenous route would likely be more direct and effective for treatment of meningitis, so future studies that may aim to optimize Carfilzomib use during GBS meningitis should aim to also optimize the drug delivery method.

Chemically targeting the V-domain of GBS BspC, and potentially other streptococcal AgI/II proteins, could prove to be useful for blocking interactions with host cell receptors and subsequent disease progression. Our results demonstrate the successful use of the virtual structure-based screening approach in identifying potential anti-virulence drugs, while also highlighting the importance of confirming hits experimentally. While the virtual screen indicated that multiple drugs scored similarly with regards to their ability to bind the BspC V-domain, only Lapatinib and Carfilzomib led to statistically significant functional impact on GBS adherence to hCMECs. There are many additional variables present in the relevant biological setting that virtual screening approaches might not consider, including pH, additional proteins that may have higher affinity to the drug, target accessibility, and drug stability to name a few. As any of these additional variables can dramatically impact drug function, experimental validation of these virtual screens is essential. It is also perhaps possible to learn from common regions of effective drugs for optimization of future drugs through rational design.

A possibility to improve this approach would be to follow up on high-ranking hits such as Lapatinib and Carfilzomib using R-group replacement chemistry to develop analogous drugs that may continue to block virulence factors such as BspC while limiting any possible negative side effects or improving efficacy. As antibiotic resistance continues to surge, the development of anti-virulence therapies will become increasingly important to replace or augment existing treatment strategies.

## Materials and methods

### Ethics statement

Animal experiments were approved by the committee on the use and care of animals at University of Colorado School of Medicine protocol #00316 and performed using accepted veterinary standards. University of Colorado School of Medicine is AAALAC accredited; and the facilities meet and adhere to the standards in the “Guide for the Care and Use of Laboratory Animals.”

### Bioinformatic analysis of *bsp* genes

BLAST was used to search for *bsp* homologs within the 135 presently available complete GBS genomes. While the serotype was already known for some of the strains containing a *bsp* gene, we used molecular serotyping to determine the serotype for any unknown strains by using BLAST to search for capsule genes that have previously been used for PCR determination of molecular serotype [67,68] **(S1 Table)**. A serotype match was determined by query coverage and pairwise identity both >99%. Three strains failed to meet the criteria for molecular serotyping and have been labeled as UD (undetermined). We used the WGS-based typing services provided by PubMLST [69] to determine the sequence type for all strains shown in **Fig 1A**.

### Bacterial strains and growth conditions

GBS clinical isolate COH1 (serotype III, ST-17) [24] and its isogenic Δ*bspC* mutant were used for most experiments. Additional GBS strains used were NEM316 (1 copy *bspA*, 3 copies *bspB*), 515 (1 copy *bspC*), and CJB111 (no *bsp* gene). All GBS strains were grown standing in THB at 37°C. *E*. *coli* strains DH5α and MC1061 were used to propagate plasmids during cloning, and *E*. *coli* BL21; pLysS was used for protein expression. All *E*. *coli* strains were grown in LB while shaking at 37°C, and GBS strains were grown in THB static at 37°C. Antibiotics were added to media for selection of strains containing plasmids at the following concentrations: 250 μg/mL erythromycin (Sigma) for pDCErm in *E*. *coli*, 5 μg/mL erythromycin (Sigma) for pDCErm in GBS, 50 μg/mL chloramphenicol for pLysS, and 100 μg/mL ampicillin for pTEV5.

### Site directed mutagenesis and construction of mutant complementation vectors

WT *bspC* was cloned into a pUC based vector (pUT18C). The Agilent QuikChange II Site-

Directed Mutagenesis Kit was used to introduce mutations. All primers are listed in **S2 Table**. Briefly, overlapping primers containing desired mutations were used with the *pfu* ultra polymerase to amplify vectors containing the mutation. The template was digested using *DpnI*, and then the vectors were transformed into *E*. *coli* DH5α and confirmed via sanger sequencing. Mutant *bspC* was then subcloned into pDCErm using EcoRI and BamHI cut sites and transformed into *E*. *coli* MC1061.

### Protein purification

Both the WT BspC V-domain and the gating loop mutant BspC V-domain proteins were purified in the same manner. WT and GLM V-domain coding sequence was cloned into the pTEV5 vector with an N-terminal His6-tag fusion and expressed in *E*. *coli* BL21; pLysS while shaking in LB at 37°C with the addition of 1 mM IPTG for 6 hours. Bacteria was pelleted and lysed with BugBuster Protein Extraction Reagent (Sigma). The lysate was centrifuged, and protein was purified from supernatant using a HIS-Select Nickel Affinity Gel Column (Sigma). The His6-tag was then cleaved using the TEV protease, and then the proteins were repurified with using the HIS-Select Nickel Affinity Gel Column.

### Protein modelling and virtual structure-based drug screen

The V-domain sequence from the COH1 *bspC* gene was input and modelled using a monomer from the previously determined structure for NEM316 BspA (PDB: 5DZ8) as a template with SWISSMODEL. All visualization of this model was done using PyMOL Version 2.0.

The BspC V-domain model was uploaded to the PLANTS docking program accessed through Cheminfo, using Lys 269 as the center of a 12-angstrom radius to screen the e-Drug3D library of FDA-approved drugs for predicted binding [33,34]. This was repeated using the *S*. *mutans* SpaP A3VP1 model (PDB: 3IPK) and TRP 431 as the center of a 12-angstrom radius.

### Circular dichroism

Circular dichroism of 1 mg/mL WT or GLM V-domain proteins was conducted using a JASCO 815 CD Spectrophotometer according to manufacturer instructions. Measurements were taken from 250–190 nm with 0.5 nm increments, 2 second digital integration time, 1 nm bandwidth, and a scanning speed of 100 nm/min.

### Microscale thermophoresis

His6-Tagged vimentin (Novus Biologicals) was labeled with RED-tris-NTA 2nd Generation dye (NanoTemper Technologies) by incubating 100 μL of vimentin (200 nM) with 100 μL of dye (100 nM) at room temperature for 30 minutes prior to a 10 minute centrifugation at 4°C. WT or GLM V-domain proteins were serially titrated and mixed at a 1:1 ratio with the labeled vimentin from a concentration of 40 μM to 0.117 μM, while vimentin was kept at a concentration of 20 nM. Measurements were performed in premium capillaries with a Monolith NT.115 Pico system at 20% excitation power.

### hCMEC culturing conditions and assays

Cells of the well-characterized human cerebral microvascular endothelial cell line (hCMEC/D3), referred to here as hCMEC were obtained from Millipore and were maintained in an EndoGRO-MV complete medium kit supplemented with 1 ng/ml fibroblast growth factor-2 (FGF-2; Millipore) [70,71]. Cells were grown at 37°C with 5% CO_2_ on surfaces collagenized using 1% rat-tail collagen.

Assays to determine the total number of cell surface-adherent bacteria were performed as described previously [12]. Briefly, bacteria were grown to mid-log phase to infect cell monolayers (1 × 10^5^ CFU, at a multiplicity of infection [MOI] of 1). Total cell-associated GBS were recovered following a 30 min incubation. Cells were detached with 0.1 mL of 0.25% trypsin-EDTA solution and lysed with addition of 0.4 ml of 0.025% Triton X-100 by vigorous pipetting. The lysates were then serially diluted and plated on THB agar to enumerate bacterial CFU relative to the input which was similarly plated immediately before infection. For protein blocking assays, hCMECs were pretreated with WT or GLM V-domain at a concentration of 10 μM per well, or the PBS vehicle control and incubated for 30 min prior to infection with GBS. After infection, assays were continued as normal for enumeration of adherent bacterial CFU.

For drug blocking assays, GBS was grown to mid-log phase, pelleted and resuspended in PBS, and then pretreated with either 1% DMSO (vehicle), 10 μM of Lapatinib, Cobicistat, Venetoclax, or Carfilzomib, or 1 μM of Paliperidone Palmitate or Tafluprost for 30 minutes at room temperature. After incubation, bacteria were normalized again by OD600 and diluted 100-fold for infection of hCMECs at an MOI of 1. After infection, drug blocking assays were continued as normal for enumeration of adherent bacterial CFU. The dose curve experiments with Lapatinib and Carfilzomib were both done in the same way as the drug blocking experiments, but with a concentration range of 0.1 nM to 10 μM for each drug treatment.

Trypan Blue dye was used to determine cytotoxicity of Lapatinib and Carfilzomib. Briefly, hCMEC monolayers grown in a 24-well plate were treated with 20 nM of each drug, DMSO (vehicle), or were left untreated for 30 minutes. The cells were then washed with PBS, and then detached with 0.1 mL of 0.25% trypsin-EDTA solution which was then diluted with 2 mL of PBS. 10 μL per well was diluted 1:10 in trypan blue and then both live and dead cells were counted using a hemocytometer.

### Antibodies and flow cytometry

The anti-BspC polyclonal antibody and an IgG antibody control (Invitrogen) were diluted to 2.28 mg/mL and adsorbed (as previously described [6]) against COH1Δ*bspC* bacteria to remove natural rabbit antibodies that react non-specifically with bacterial surface antigens by incubating with COH1Δ*bspC* overnight at 4°C, with rotation. Bacteria were pelleted by centrifugation and the supernatant was collected and filtered using 0.22 μM cellulose acetate SpinX centrifuge tube filters (Costar).

In order to assess surface expression of WT or mutant BspC, GBS strains were grown to OD600 of 0.25 in EndoGRO-MV culture medium (Millipore) to mimic host infection conditions, pelleted by centrifugation, and resuspended in PBS. Approximately, 1 x 10^6^ CFU of each strain was incubated with either adsorbed anti-BspC antibody or adsorbed anti-rabbit IgG at a 1:50 dilution at 4°C, overnight, with rotation. The next day, bacteria were washed via centrifugation, and labeled with a donkey anti-rabbit IgG conjugated to AlexaFluor488 (Invitrogen) at a 1:2,000 dilution for 45 minutes at room temperature with rotation. Samples were washed again and then resuspended and read on a FACScalibur flow cytometer (BD Biosciences) and analyzed using FlowJo (v10) software.

### Mouse model of hematogenous GBS meningitis

We utilized a mouse GBS infection model as described previously [6,12,30,31]. Briefly, 8-week-old male CD-1 mice (Charles River) were injected intravenously with ~4 × 10^8^ CFU of COH1Δ*bspC* containing pDCErm (either empty vector or expressing WT or mutant *bspC*). Mice were euthanized 48 hours post infection and blood and brain tissue were collected. The brain tissue was homogenized and plated on THA agar for enumeration of bacterial CFU along with blood, as well as THA supplemented with 5 μg/mL Erythromycin to confirm plasmid retention.

For Carfilzomib treatment experiments, 8-week-old male CD-1 mice were first injected with either 100 μL of Carfilzomib dissolved in sesame oil and 10% DMSO (2.5 mg/kg) or the vehicle control intraperitoneally. This dose was chosen based on other carfilzomib mouse treatment studies that generally have used between 1.5–5 mg/kg [72–75]. Two hours later, the mice were injected intravenously with 8.5 × 10^8^ CFU of WT or Δ*bspC* COH1. Every 24 hours after the initial treatment, an additional dose was administered until the mice were euthanized 72 hours post infection and blood and brain tissue were collected. The brain tissue was homogenized and plated on THA agar for enumeration of bacterial CFU along with blood.

### Histology

Mouse brain tissue fixed in neutral buffered formalin at room temperature for three days prior to being embedded in paraffin and sectioned. Sections were stained using hematoxylin and eosin and two images per section were taken using a BZ-X710 microscope (Keyence). Each image was analyzed by ImageJ to quantify meningeal thickness at three representative points per image.

## Supporting information

S1 Fig Bsp Distribution and Conservation. **(A)** The amino acid sequences of each *bsp* type (A-D) were individually aligned using MUSCLE. The consensus sequences for each type are shown. **(B)** The amino acid sequences for all *bsp* genes found from the currently available completed GBS genomes were aligned using MUSCLE. **(C)** The amino acid sequences of the V-domain from all *bspC* genes found from the currently available completed GBS genomes were aligned using MUSLCE. For both (B) and (C), any colors within the sequences indicate a disagreement from the consensus sequence (top). Protein domains are annotated under the consensus sequence. The percent identity at each site is shown under the domain annotations, where green indicates 100% conservation.
(TIFF)

S2 Fig Validation of BspC Mutant Complement Strains. **(A)** Growth curve of indicated strains grown in THB supplemented with 5 μg/mL Erythromycin. **(B)** The percentage of GBS cells that contained surface expressed BspC WT and mutant proteins was determined using FlowJo. (C,D) Mice were infected with ~4x10^8^ WT or Δ*bspC* containing the pDCErm empty vector, or the Δ*bspC*; p*bspC* complement strain. The GBS CFU counts from brain **(C)** and blood **(D)** after 48 hours are shown. Brain tissue **(E)** and blood **(F)** from Fig 4 were simultaneously plated on THA and THA supplemented with 5 μg/mL Erythromycin to confirm plasmid retention. Brain tissue **(G)** and blood **(H)** from Fig 5 were simultaneously plated on THA and THA supplemented with 5 μg/mL Erythromycin to confirm plasmid retention. Statistical analysis: (B) One-way ANOVA with Dunnett’s multiple comparisons, (C-H) Two-way ANOVA with Tukey’s multiple comparisons.
(TIFF)

S3 Fig Characterization of Drug Effects. **(A)** OD600 of WT COH1 grown in THB supplemented with a range of concentrations of the indicated drugs for 24 hours. **(B)** GBS Δ*bspC* was pretreated with DMSO (vehicle), 10 μM of Lapatinib, Cobicistat, Venetoclax, or Carfilzomib, or 1 μM of Paliperidone Palmitate or Tafluprost 30 minutes prior to infection of hCMECs. CFU were plated to assess blocking of GBS adherence after 30 minutes of incubation. **(C)** hCMECs were treated with vehicle or 20 nM Lapatinib or Carfilzomib for 30 minutes. Trypan blue staining was used to measure hCMEC survival relative to an untreated control. **(D)** hCMECs were treated with vehicle, 20 nM Lapatinib, or Carfilzomib for 30 minutes and then washed once with PBS to remove excess drugs prior to infection. CFU were plated to assess GBS adherence after 30 minutes of incubation. **(E)** GBS was pretreated with either DMSO, Lapatinib, or Carfilzomib at the indicated concentrations 30 minutes prior to infection of hCMECs. CFU were plated to assess blocking of GBS adherence after 30 minutes of incubation. The dashed line indicates the mean adherence of the *ΔbspC* mutant pretreated with DMSO. A, C, and D display representative data from one of two independent experiments, where error bars indicate the standard deviation. B and E display pooled data from three independent experiments, where error bars indicate the standard error of the mean. **(F)** Top 10 hits from the PLANTS virtual structure-based screen of e-Drug3D library of FDA approved drugs against the *S*. *mutans* SpaP V-domain. **(G)** The virtual structure-based screen shown in **F** yielded Carfilzomib as a top-ten hit. A model of Carfilzomib bound to the *S*. *mutans* SpaP V-domain pocket is shown. Visualization done using PyMOL. Statistical analysis: (C and D) One-way ANOVA with Tukey’s multiple comparisons, (B and E) Two-way ANOVA with Tukey’s multiple comparisons.
(TIFF)

S4 Fig Carfilzomib Blocks Bsp Dependent Adherence of GBS to hCMECs. GBS strains NEM316 (*bspA*, *bspB*) **(A)**, 515 (*bspC*) **(B)**, and CJB111 (no *bsp* gene) **(C)** were pretreated with DMSO (vehicle) or 10 μM of Carfilzomib 30 minutes prior to infection of hCMECs. CFU were plated to assess blocking of GBS adherence after 30 minutes of incubation. Pooled data from three independent experiments is shown. Error bars indicate standard error of the mean. Statistical analysis: (A-C) Unpaired t tests. *, P < 0.05; ***, P < 0.0005.
(TIFF)

S1 Table Specific genes used for molecular serotyping. (DOCX)

S2 Table Primers used in this study. (DOCX)
