# Supplementary figures and images for: Targeting the BspC-vimentin interaction to develop anti-virulence therapies during Group B streptococcal meningitis
Source: PLoS Pathog. 2022 Mar 22;18(3):e1010397. doi: 10.1371/journal.ppat.1010397 (PMC8939794; doi:10.1371/journal.ppat.1010397)

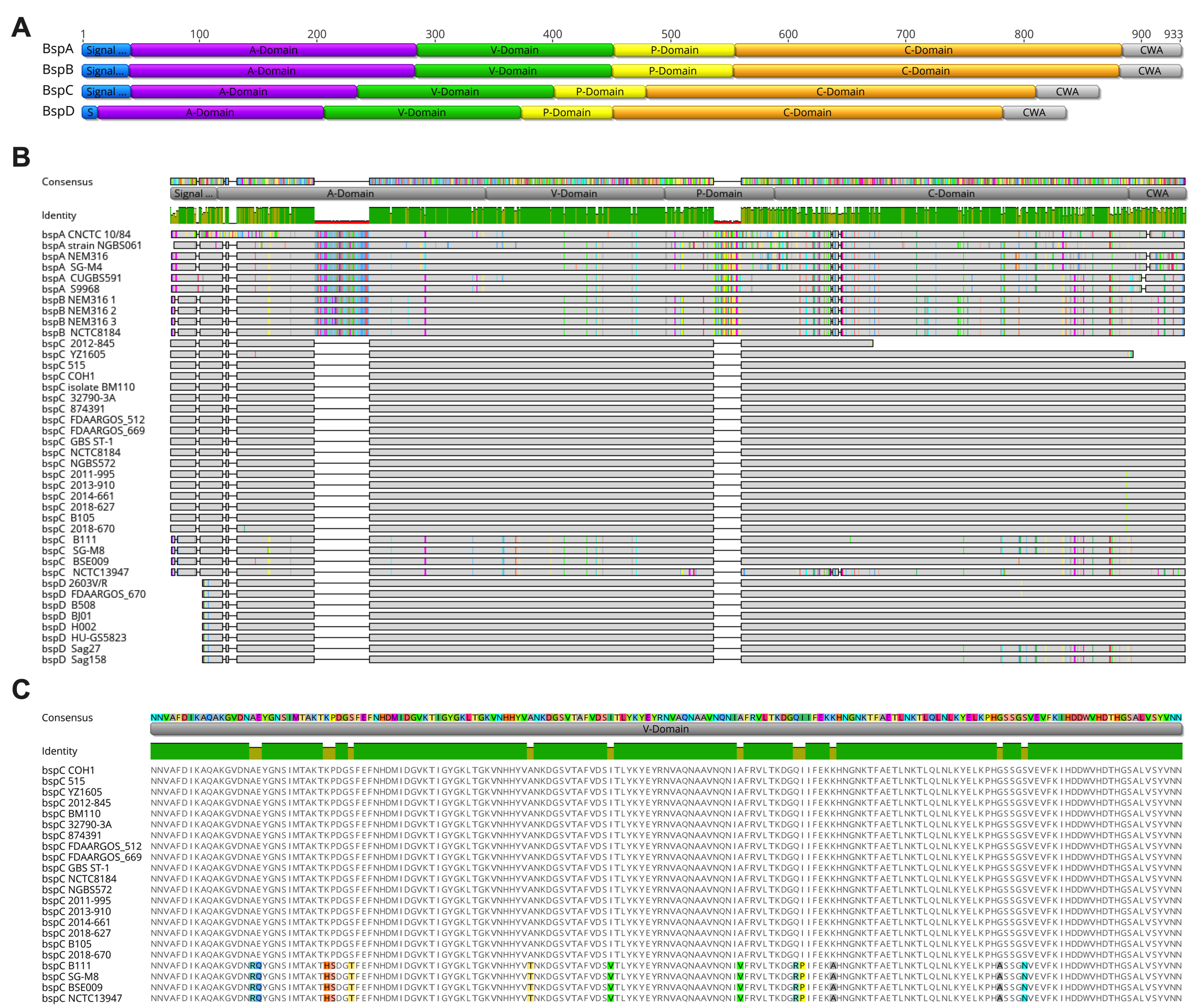

Supplement: S1 Fig — (A) The amino acid sequences of each bsp type (A-D) were individually aligned using MUSCLE. The consensus sequences for each type are shown. (B) The amino acid sequences for all bsp genes found from the currently available completed GBS genomes were aligned using MUSCLE. (C) The amino acid sequences of the V-domain from all bspC genes found from the currently available completed GBS genomes were aligned using MUSLCE. For both (B) and (C), any colors within the sequences indicate a disagreement from the consensus sequence (top). Protein domains are annotated under the consensus sequence. The percent identity at each site is shown under the domain annotations, where green indicates 100% conservation. (TIFF) [file ppat.1010397.s001.tiff]

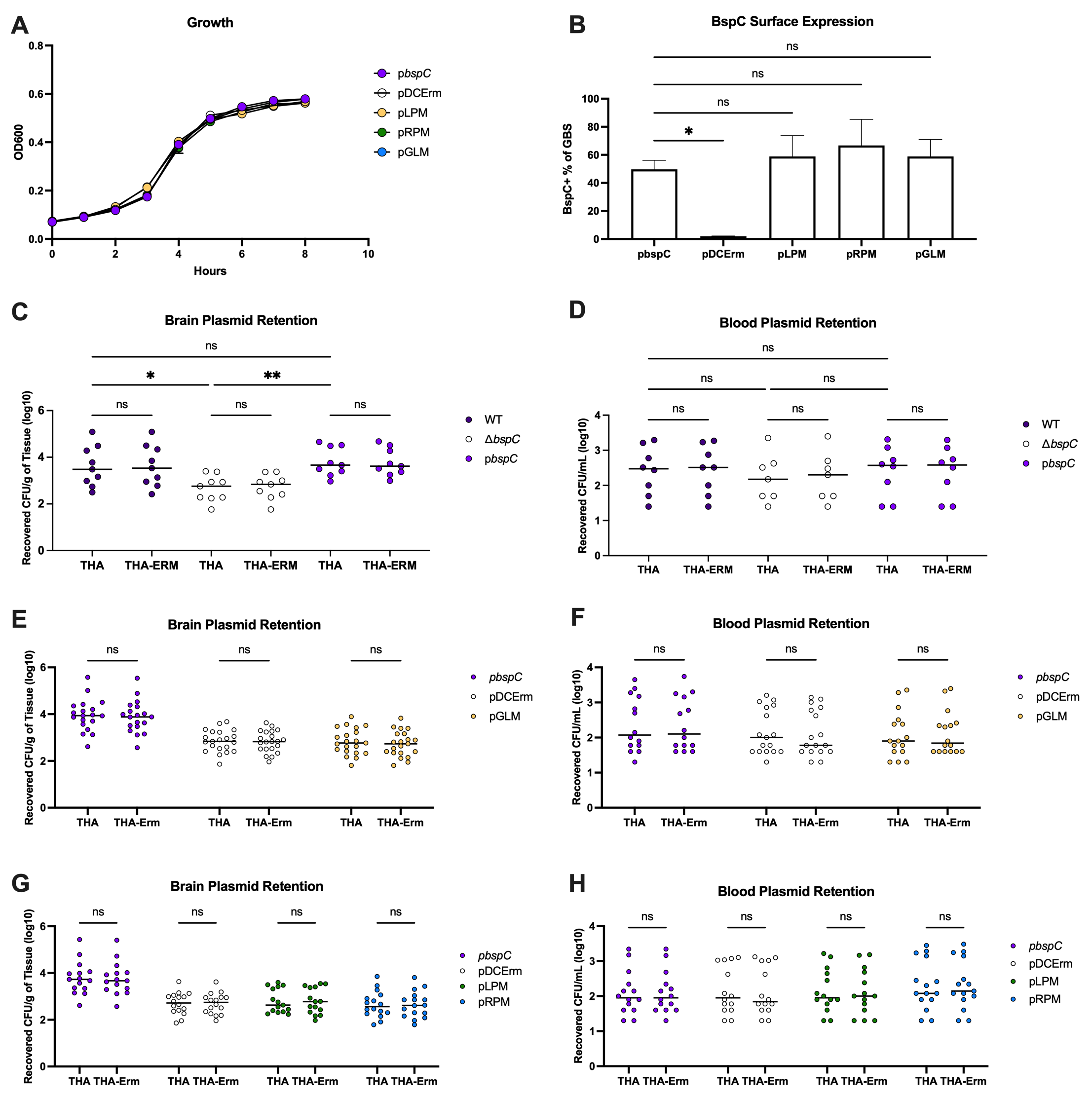

Supplement: S2 Fig — (A) Growth curve of indicated strains grown in THB supplemented with 5 μg/mL Erythromycin. (B) The percentage of GBS cells that contained surface expressed BspC WT and mutant proteins was determined using FlowJo. (C,D) Mice were infected with ~4x10^8^ WT or ΔbspC containing the pDCErm empty vector, or the ΔbspC; pbspC complement strain. The GBS CFU counts from brain (C) and blood (D) after 48 hours are shown. Brain tissue (E) and blood (F) from Fig 4 were simultaneously plated on THA and THA supplemented with 5 μg/mL Erythromycin to confirm plasmid retention. Brain tissue (G) and blood (H) from Fig 5 were simultaneously plated on THA and THA supplemented with 5 μg/mL Erythromycin to confirm plasmid retention. Statistical analysis: (B) One-way ANOVA with Dunnett’s multiple comparisons, (C-H) Two-way ANOVA with Tukey’s multiple comparisons. (TIFF) [file ppat.1010397.s002.tiff]

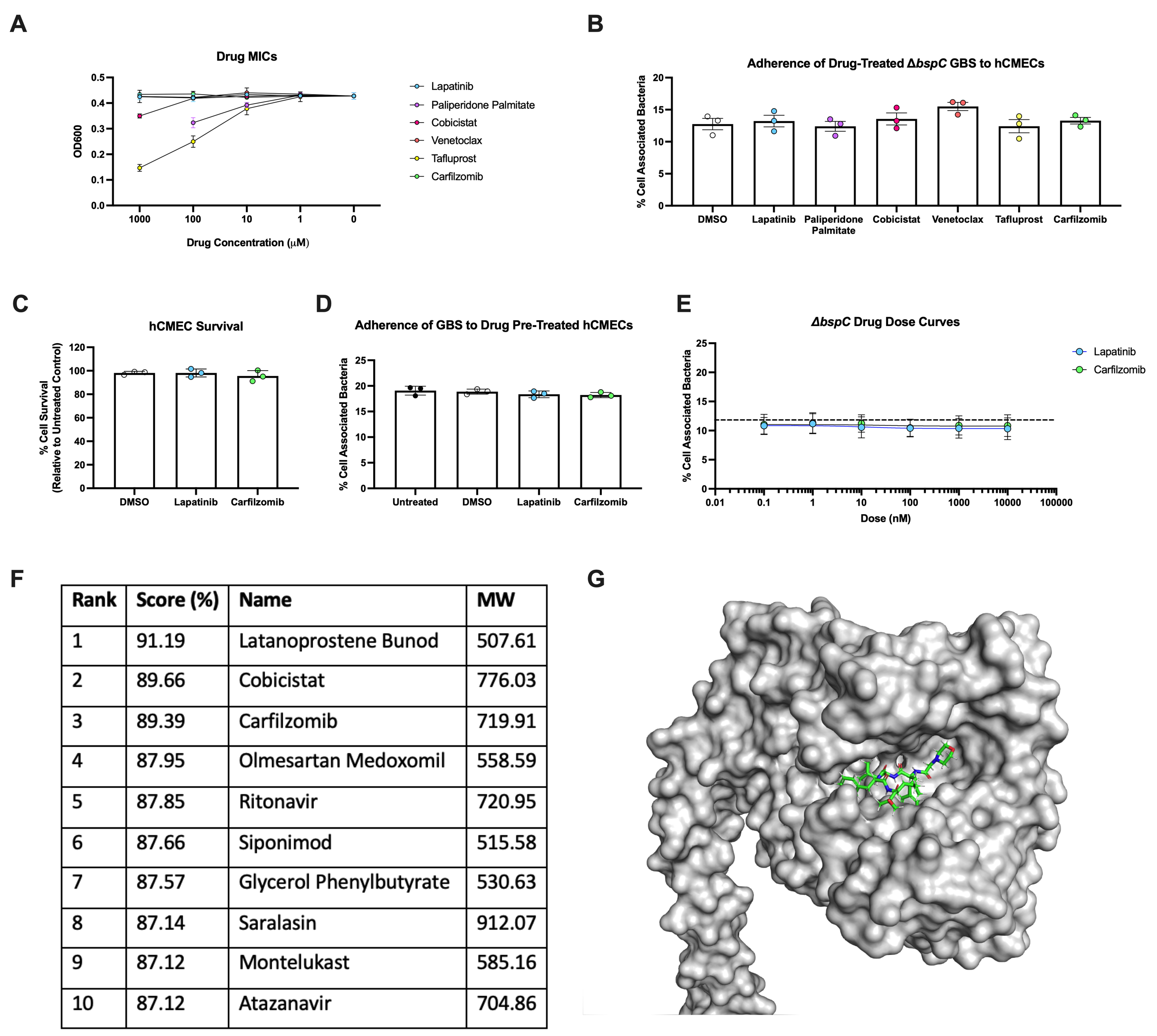

Supplement: S3 Fig — (A) OD600 of WT COH1 grown in THB supplemented with a range of concentrations of the indicated drugs for 24 hours. (B) GBS ΔbspC was pretreated with DMSO (vehicle), 10 μM of Lapatinib, Cobicistat, Venetoclax, or Carfilzomib, or 1 μM of Paliperidone Palmitate or Tafluprost 30 minutes prior to infection of hCMECs. CFU were plated to assess blocking of GBS adherence after 30 minutes of incubation. (C) hCMECs were treated with vehicle or 20 nM Lapatinib or Carfilzomib for 30 minutes. Trypan blue staining was used to measure hCMEC survival relative to an untreated control. (D) hCMECs were treated with vehicle, 20 nM Lapatinib, or Carfilzomib for 30 minutes and then washed once with PBS to remove excess drugs prior to infection. CFU were plated to assess GBS adherence after 30 minutes of incubation. (E) GBS was pretreated with either DMSO, Lapatinib, or Carfilzomib at the indicated concentrations 30 minutes prior to infection of hCMECs. CFU were plated to assess blocking of GBS adherence after 30 minutes of incubation. The dashed line indicates the mean adherence of the ΔbspC mutant pretreated with DMSO. A, C, and D display representative data from one of two independent experiments, where error bars indicate the standard deviation. B and E display pooled data from three independent experiments, where error bars indicate the standard error of the mean. (F) Top 10 hits from the PLANTS virtual structure-based screen of e-Drug3D library of FDA approved drugs against the S. mutans SpaP V-domain. (G) The virtual structure-based screen shown in F yielded Carfilzomib as a top-ten hit. A model of Carfilzomib bound to the S. mutans SpaP V-domain pocket is shown. Visualization done using PyMOL. Statistical analysis: (C and D) One-way ANOVA with Tukey’s multiple comparisons, (B and E) Two-way ANOVA with Tukey’s multiple comparisons. (TIFF) [file ppat.1010397.s003.tiff]

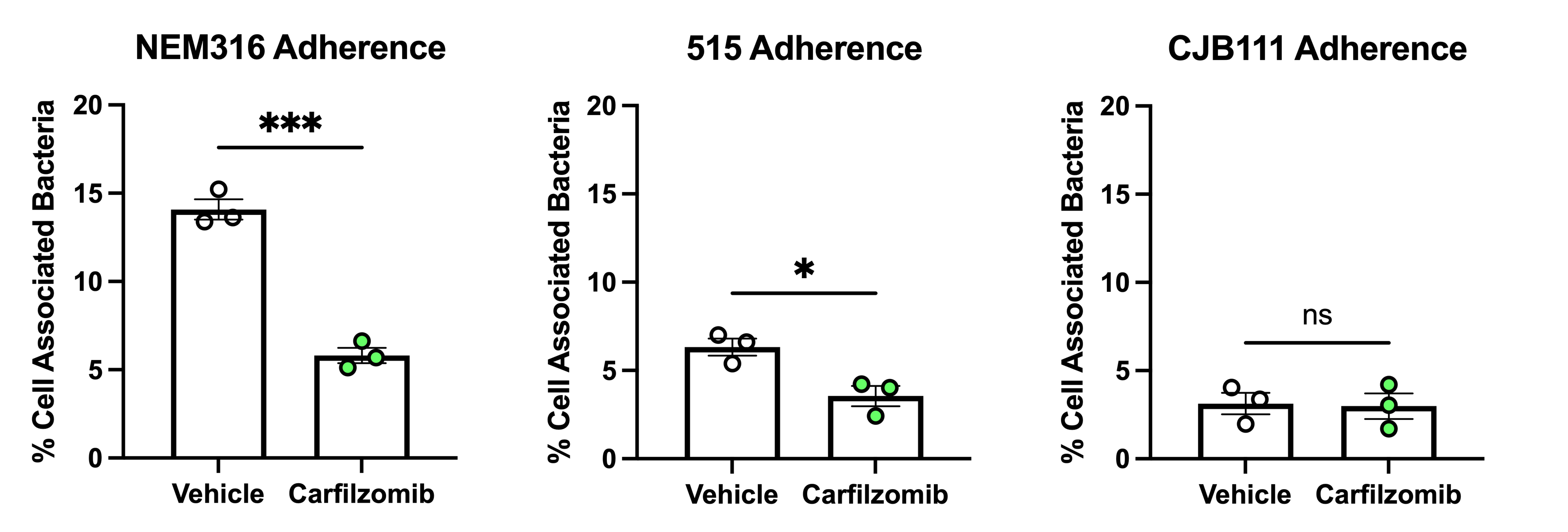

Supplement: S4 Fig — GBS strains NEM316 (bspA, bspB) (A), 515 (bspC) (B), and CJB111 (no bsp gene) (C) were pretreated with DMSO (vehicle) or 10 μM of Carfilzomib 30 minutes prior to infection of hCMECs. CFU were plated to assess blocking of GBS adherence after 30 minutes of incubation. Pooled data from three independent experiments is shown. Error bars indicate standard error of the mean. Statistical analysis: (A-C) Unpaired t tests. *, P < 0.05; ***, P < 0.0005. (TIFF) [file ppat.1010397.s004.tiff]
