# Supplementary material for: Targeting the BspC-vimentin interaction to develop anti-virulence therapies during Group B streptococcal meningitis
Source: PLoS Pathog. 2022 Mar 22;18(3):e1010397. doi: 10.1371/journal.ppat.1010397 (PMC8939794; doi:10.1371/journal.ppat.1010397)
Supplement: S1 Table — (DOCX) [file ppat.1010397.s005.docx]

**S1 Table:** Specific genes used for molecular serotyping [1,2].

| **Serotype** | **Gene Targets** | **Genbank** |
| --- | --- | --- |
| Ia | *cps1aH* | AB028896 |
| Ib | *cps1bJ/K* | AB050723 |
| II | *cps2K* | AY375362 |
| III | *Cps1a/2/3I/3J* | AF163833 |
| IV | *cps4N* | AF355776 |
| V | *csp5O* | AF349539 |
| VI | *cps6I* | AF337958 |
| VII | *cps7M* | AY376403 |
| VIII | *cps8J* | AY375363 |
| IX | *Cps9H/M* | LT671992 |

References

1. Poyart C, Tazi A, Reglier-Poupet H, Billoet A, Tavares N, Raymond J, et al. Multiplex PCR assay for rapid and accurate capsular typing of group B streptococci. J Clin Microbiol. 2007;45(6):1985-8.

2. Slotved HC, Kong F, Lambertsen L, Sauer S, Gilbert GL. Serotype IX, a Proposed New Streptococcus agalactiae Serotype. J Clin Microbiol. 2007;45(9):2929-36.
