# Supplementary material for: Targeting the BspC-vimentin interaction to develop anti-virulence therapies during Group B streptococcal meningitis
Source: PLoS Pathog. 2022 Mar 22;18(3):e1010397. doi: 10.1371/journal.ppat.1010397 (PMC8939794; doi:10.1371/journal.ppat.1010397)
Supplement: S2 Table — (DOCX) [file ppat.1010397.s006.docx]

**S2 Table:** Primers used in this study.

| **Primer Sequence (5’-3’)** | **Notes** |
| --- | --- |
| ATATGGATCCATGGATCAAGTTACAACTCAAG | Fwd Cloning BspC into pUT18C |
| ATATGAATTCTTATCCTTTAGCAGGAGCAA | Rev Cloning BspC into pUT18C |
| GGTGTTGATAACCCTGAATATGGAAACTCAATCATGACTCCAAAAACTAAACC | Fwd pUT18C::BspC Mutagenesis A250P/A259P |
| GGTTTAGTTTTTGGAGTCATGATTGAGTTTCCATATTCAGGGTTATCAACACC | Rev pUT18C::BspC Mutagenesis A250P/A259P |
| GCAAAAACTAAACCTGACGGAAGTGCCGAGGCTAACGCTGATATGATCGATG | Fwd pUT18C::BspC Mutagenesis FxFxH to AxAxA |
| CATCGATCATATCAGCGTTAGCCTCGGCACTTCCGTCAGGTTTAGTTTTTGC | Rev pUT18C::BspC Mutagenesis FxFxH to AxAxA |
| AAGCGTTGAAGTCGCTGAGATTGCTGATGACGCGGTACATGACAC | Fwd pUT18C::BspC Mutagenesis FKxHxW to AExAxA |
| GTGTCATGTACCGCGTCATCAGCAATCTCAGCGACTTCAACGCTT | Rev pUT18C::BspC Mutagenesis FKxHxW to AExAxA |
| ATATGAATTCATGTATAAAAATCAAAACACAAAAGG | Fwd Cloning BspC into pDCERM |
| ATATGGATCCTTAATTTTCTTTGCGTTTTTTAAATC | Rev Cloning BspC into pDCERM |
| TGGAATTCTCGAGCTCCCGGAATGTTGCTTTTGATATCAAAG | Fwd Gibson Cloning BspC V-Domain into pTEV5 |
| GTTATTGCTCAGCGGCCGCGTTAATTATTAACATAAGACACTAAAGC | Rev Gibson Cloning BspC V-Domain into pTEV5 |
